# Evaluating Accuracy of DNA Pool Construction Based on White Blood Cell Counts

**DOI:** 10.3389/fgene.2021.635846

**Published:** 2021-02-05

**Authors:** Amy N. Abrams, Tara G. McDaneld, John W. Keele, Carol G. Chitko-McKown, Larry A. Kuehn, Michael G. Gonda

**Affiliations:** ^1^Department of Animal Science, South Dakota State University, Brookings, SD, United States; ^2^Department of Animal Science, Berry College, Mount Berry, GA, United States; ^3^Genetics, Breeding, and Animal Health Research Unit, U.S. Meat Animal Research Center, USDA-ARS, Clay Center, NE, United States; ^4^Animal Health Genomics Research Unit, U.S. Meat Animal Research Center, USDA-ARS, Clay Center, NE, United States

**Keywords:** bovine, pooling, genotyping, white blood cells, DNA quantification

## Abstract

Pooling individual samples prior to DNA extraction can mitigate the cost of DNA extraction and genotyping; however, these methods need to accurately generate equal representation of individuals within pools. The objective of this study was to determine accuracy of pool construction of blood samples based on white blood cell counts compared to two common DNA quantification methods. Fifty individual bovine blood samples were collected, and then pooled with all individuals represented in each pool. Pools were constructed with the target of equal representation of each individual animal based on number of white blood cells, spectrophotometric readings, spectrofluorometric readings, and whole blood volume with 9 pools per method and a total of 36 pools. Pools and individual samples that comprised the pools were genotyped using a commercially available genotyping array. ASReml was used to estimate variance components for individual animal contribution to pools. The correlation between animal contributions between two pools was estimated using bivariate analysis with starting values set to the result of a univariate analysis. Adonis test on distance matrix from the animal correlation showed clustering with method, and higher correlations between methods than within (*P* < 1 × 10^–6^). White blood cell count was predictive of sample representation when compared to pooling based on DNA concentration. Therefore, constructing pools using white blood cell counts prior to DNA extraction may reduce cost associated with DNA extraction and genotyping and improve representation of individuals in a pool.

## Introduction

Determining the genetic basis of complex traits requires genotyping a large number of individuals in order to achieve reliable and replicable findings. While the use of genotyping panels with hundreds of thousands of single nucleotide polymorphisms (SNPs) has provided the capability to scan genomic regions for genetic markers associated with a trait or disease, the cost of these studies can be prohibitive. Pooling genomic DNA samples offers a way to substantially reduce the cost of large-scale genotyping studies, providing an economic approach to investigate the genetic bases for many traits and diseases (Macgregor et al., 2008). This approach replaces individual genotyping with genotyping of pooled genomic DNA and has been successfully applied in multiple case-control association and QTL mapping studies (Huang et al., 2010; McDaneld et al., 2014; Strillacci et al., 2014). Additionally, many phenotypic records are collected at the commercial level but are not included in genomic evaluations because it is not economically feasible to genotype individual commercial animals. Pooling DNA samples provides a means to incorporate phenotypes from commercial animals into genetic evaluations (Reverter et al., 2016; Bell et al., 2017; Baller et al., 2020). Pooling genomic DNA samples captures greater than 80% of the power of individual genotyping by utilizing allele frequency estimations from pooled DNA samples to identify regions of association that can be targeted for further investigation (Barratt et al., 2002; Macgregor et al., 2008).

Detection of true regions of association using pooled DNA methods is influenced by variance in allele frequency estimates resulting from quantitative errors introduced at different stages of the experimental process (Barratt et al., 2002). One such source of error can occur during DNA quantification and pool construction. Previous research has demonstrated disagreement and inconsistency between prominent DNA quantification methods, including spectrofluorometry and spectrophotometry (Holden et al., 2009; Li et al., 2014; Yu et al., 2017).

While DNA pooling has made large scale association studies more feasible, pooling samples prior to DNA extraction could further mitigate the cost of DNA extraction and therefore genotyping. Craig et al. (2009) demonstrated that pooling whole blood samples prior to DNA extraction substantially reduced the time, cost, and labor required for large-scale genotyping studies. Pooling samples prior to DNA extraction has also been successful using pooled milk samples to attempt to discover QTL affecting milk protein percentage and pooling bovine liver and lung tissue samples to study the genetics of susceptibility to liver abscesses and lung lesions in cattle (Lipkin et al., 1998; Mosig et al., 2001; Keele et al., 2015, 2016). Blood samples are a commonly collected sample in the livestock industry and are relatively simple and inexpensive to obtain. Because white blood cells contain equal amounts of DNA, and are the main source of DNA in whole blood, pooling samples based on equal white blood cell counts should result in an equal contribution from each individual sample DNA within a pool. Furthermore, because individual samples are added to the pool based on white blood cell count rather than DNA concentration, the variation within pools may actually be lower compared to pools constructed from fluorometric or photometric quantification methods if cell counts are more accurate than these DNA quantification methods. Therefore, the objective of this study was to determine variation in representation of animals within pools constructed based on white blood cell counts.

## Materials and Methods

### Sample Collection

All animal use was approved by the U.S. Meat Animal Research Center (USMARC) Institutional Animal Care and Use Committee. Samples were collected from 50 steers from advanced generations of the USMARC Germplasm Evaluation Program (GPE) at the USMARC feedlot in Clay Center, Nebraska. The cattle were diverse crossbred steers with recent ancestry in the last three generations including Black Angus, Red Angus, Hereford, Shorthorn, Santa Gertrudis, Limousin, Charolais, Braunvieh, Salers, Tarentaise, and Simmental in order of contribution (Schiermiester et al., 2015). All animals were healthy based on normal cell count range. Blood samples were collected via jugular venipuncture into 9-ml Sarstedt Monovette blood collection tubes containing ethylenediaminetetraacetic acid (EDTA) as an anticoagulant (Sarstedt AG & Co., Numbrecht, Germany).

### Sample Processing and Pool Construction

**FIGURE 1:**
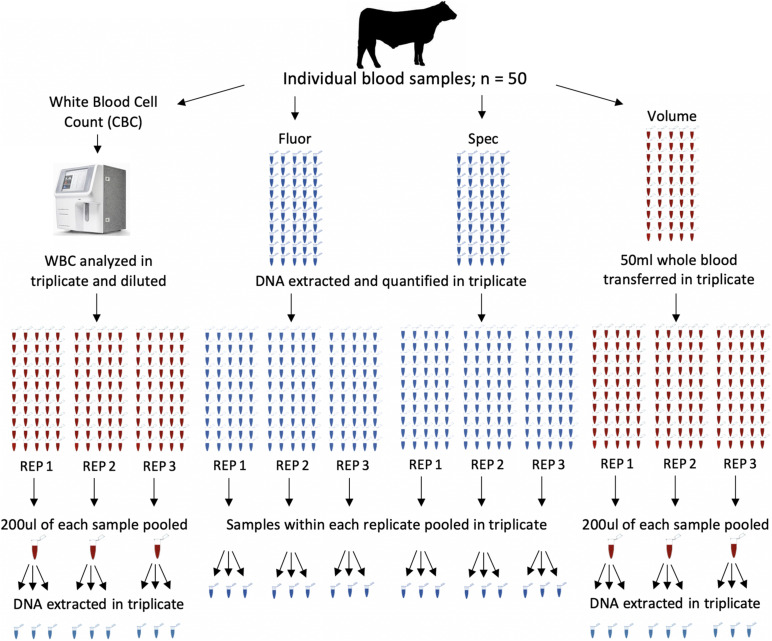
Pool construction for four different methods, each with fifty individuals represented in each pool. Samples from each method were pooled and extracted in triplicate for a total of 36 pools. The four methods of pooling were based on equal amounts of white blood cell counts (CBC), DNA concentrations determined by spectrofluorometer (Fluor), DNA concentrations determined by spectrophotometer (Spec), and whole blood based on volume (Volume). Each main column represents a method of pooling. Columns of tubes within a method represent samples going into each pool. Red tubes are blood and blue tube are extracted DNA. Pools were constructed with all 50 individuals represented in each pool. Pools were constructed in triplicate and DNA was extracted from each pool in triplicate.

DNA pools were constructed from all 50 animals using four different methods. Nine pools were constructed for each DNA pooling method, for a total of 36 pools (Figure 1). The four methods of pooling were based on equal amounts of (1) white blood cells (CBC), (2) DNA concentrations determined by spectrofluorometer (Fluor), (3) DNA concentrations determined by spectrophotometer (Spec), and (4) whole blood based on volume (Volume). The same 50 animals were used in each pool across all pooling methods.

### Pool Construction Based on White Blood Cell Count

One ml of whole blood with EDTA was transferred to a 2-ml screw cap vial and mixed continuously prior to white blood cell count using an Element HT5 Veterinary Hematology Analyzer (Heska, Loveland, CO, United States). Samples were analyzed in triplicate. Based on the cell count for each sample replicate (3 per sample), whole blood samples were diluted in phosphate buffer solution (PBS) to obtain white blood cell concentrations of 5.0 × 10^3^ in a total volume of 200 μL. This resulted in a total of 150 dilutions, 3 dilutions for each sample. Dilutions within each replicate were then pooled, with all fifty samples pooled based on the first, second reading, and third reading, resulting in 3 pools (CBC1, CBC2, and CBC3). Diluted pools were frozen at −20°C prior to DNA extraction. After thawing and mixing, equal volumes (200 μL) of the diluted blood pools were extracted in triplicate using the QIAamp DNA Mini Kit following the manufacturer’s instructions (Qiagen, Santa Clarita, CA, United States). As a result, nine diluted blood pools based on WBC were extracted (CBC 1–1, CBC 1–2, CBC 1–3, CBC 2–1, CBC 2–2, CBC 2–3, CBC 3–1, CBC 3–2, and CBC 3–3). Quality of DNA was evaluated for all samples using gel electrophoresis to ensure high molecular weight DNA was present and intact.

### Pool Construction Based on Whole Blood Volume

Whole blood pools were then generated by combining 50 μL of whole blood with EDTA from all 50 individuals to a pool. This was completed in triplicate, resulting in three pools (Volume1, Volume2, and Volume3). Volume pools and individual whole blood samples were frozen at −20°C prior to DNA extraction. After thawing and mixing, equal volumes (200 μL) of the three volume pools were extracted in triplicate using previously described methods, resulting in nine pools (Volume 1–1, Volume 1–2, Volume 1–3, Volume 2–1, Volume 2–2, Volume 2–3, Volume 3–1, Volume 3–2, and Volume 3–3).

### Pool Construction Determined by Spectrofluorometer

After thawing and mixing, equal volumes (200 μL) of whole blood from each individual was extracted using previously described methods. Extracted DNA was mixed and quantified in triplicate using the Quantifluor^®^ dsDNA System following the manufacturer’s instructions (Promega, Madison, WI, United States). Quantification was completed using the DeNovix DS-11 FX + Spectrophotometer/Fluorometer using the Quantifluor dsDNA fluorometric setting. Based on the spectrofluorometer readings within each replicate, samples were then pooled by equal concentrations, with all fifty samples pooled based on the first, second reading, and third reading, in triplicate, resulting in nine pools (Fluor 1–1, Fluor 1–2, Fluor 1–3, Fluor 2–1, Fluor 2–2, Fluor 2–3, Fluor 3–1, Fluor 3–2, and Fluor 3–3).

### Pool Construction Determined by Spectrophotometer

The DNA extracted from whole blood, as described previously for the spectrofluorometer, was also quantified in triplicate by the DeNovix DS-11 FX + Spectrophotometer/Fluorometer (DeNovix Inc., Wilmington, DE, United States) using 2 μL of sample and the dsDNA photometric setting. Based on the spectrophotometer readings within each replicate, samples were then pooled by equal concentrations, with all fifty samples pooled based on the first reading, the second reading, and the third reading, in triplicate, resulting in nine pools (Spec 1–1, Spec 1–2, Spec 1–3, Spec 2–1, Spec 2–2, Spec 2–3, Spec 3–1, Spec 3–2, and Spec 3–3).

### Genotyping

All individual animals and pools were genotyped with the Illumina Bovine GGP 50K SNP array (47,843 SNP; Illumina, Inc., San Diego, CA, United States) by Neogen Corporation (Lincoln, NE, United States).

### Statistical Analysis

Pools with smaller variance components for animal contributions provide more accurate information for genetic evaluation. High correlations between pools constructed from the same animals indicate consistency in pipetting, DNA extraction efficiency, and homogeneity of DNA in the pooled samples. ASReml in R (Butler et al., 2017; R Core Team, 2019) was used to estimate variance components for animal contribution to pools using pooling allele frequency as the dependent variable and number of copies of A allele (individual animal genotypes) divided by 2 for the 50 individually genotyped animals contributing to the pools as the independent variable as previously reported in McDaneld et al. (2014). Animal genotypes were random covariates in a bivariate analysis, using 2 pools at a time for computational feasibility. The correlation between animal contributions to 2 pools was estimated using bivariate analysis with starting values set to the result of a univariate analysis. From the 34 pools analyzed there were 561 (34 × 33/2) bivariate analyses.

## Results

Preliminary analyses indicated overrepresentation of 3 animals in CBC 3–3 and 1 animal in Fluor 3–3. Examination of location of these 2 pools indicated that the overrepresented animals in both cases were in adjacent wells. This result suggested that the pools were cross-contaminated by individual animals in adjacent wells during DNA transfer to the plate, transit or sample transfer during genotyping. To verify cross contamination in the original plate, 3 additional aliquots of the original CBC blood pools were extracted and genotyped and no evidence of over-represented animals was identified. Because the reconstructed CBC pools confirmed that this was a case of cross contamination rather than overrepresentation, it was not necessary to additionally reconstruct the Fluor pools. Based on these findings, CBC 3–3 and Fluor 3–3 were eliminated from further analyses due to cross-contamination. This resulted in 8 CBC, 8 Fluor, 9 Spec, and 9 Volume pools.

**FIGURE 2:**
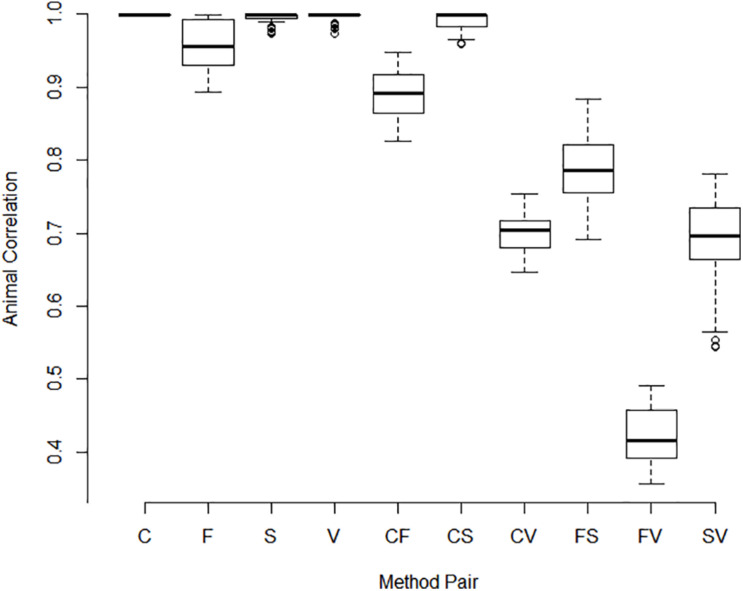
Correlations between pools for animal contributions by method of pooling. The correlation between animal contributions to 2 pools was estimated using bivariate analysis with starting values set to the result of a univariate analysis. Methods include equal volume of blood diluted to constant white blood cell count (C), equal DNA by spectrofluorometry (F), equal DNA by spectrophotometry (S), and equal volume of whole blood (V). Single letter codes designate correlations between pools within method whereas two letter codes signify correlations between pools constructed by different methods. Correlations among animal contributions within method were near 1 and were lower between pools of different methods. Pools constructed based on separate measurements within pool or replicated pool constructions and or extractions were highly correlated as evidenced by the small interquartile distance.

**FIGURE 3:**
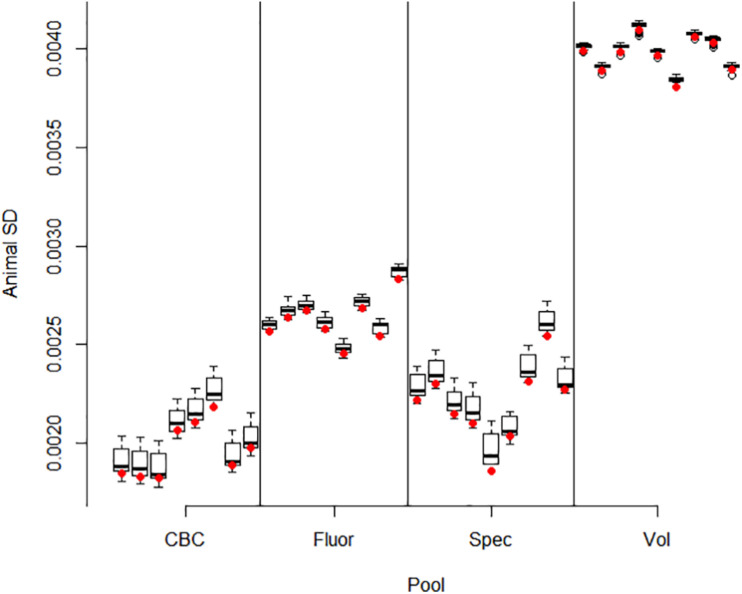
Animal contribution SD by pool. Methods include equal volume of blood diluted to constant white blood cell count (CBC), equal DNA by spectrofluorometry (Fluor), equal DNA by spectrophotometry (Spec), and equal volume of whole blood (Vol). Bivariate ASReml analyses were conducted for all 561 pairs of 34 pools. The variance component for animal contribution was estimated for each pool in combination with all 33 other pools. The restricted maximum likelihood estimate varied slightly by each pairing as evidenced by the interquartile range. The red dot gives the animal SD for the univariate analyses which were used as starting values in the bivariate analyses. In general, information from the paired pool resulting from correlation information had little impact and small interquartile difference and ranges in estimates for a given pool.

Correlations between pools for animal contributions estimated by 561 bivariate ASReml analyses are shown in Figure 2. Higher dimensional (>2) multivariate analyses were not run because of difficulty of ASReml to converge within the parameter space, presumably because of high correlations among pools within method. Adonis test on distance matrix from the animal correlation showed clustering with method, and higher correlations between methods than within (*P* < 1 × 10^–6^). Animal contribution standard deviation (SD) estimated by 34 univariate and 561 bivariate ASReml analyses are shown in Figure 3. Animal contribution SD varied by method (*P* = 2.39 × 10^–6^) based on Kruskal Wallis sum-rank test. Pools constructed based on CBC and Spec methods resulted in similar SD and both were lower compared to the Fluor pooling method. As expected, the volume pools resulted in the greatest SD because of differences in cell count and DNA concentration among individual samples.

## Discussion

Pooling samples prior to DNA extraction could mitigate the cost of genotyping further if these methods can accurately generate equal representation of individuals within pools. Based on the outcome of this study, white blood cell count is a viable approach for pool construction and was either equal or more predictive of sample representation compared to other pooling methods evaluated. Current DNA quantification methods have proven adequate to generate pools for genotyping (McDaneld et al., 2014; Keele et al., 2015). However, the pools constructed from CBC had similar sample representation of DNA from each individual compared to Spec pools and were more likely to have equal sample representation compared to Fluor and Volume pooling methods.

Because variation in animal contribution was different between methods, with CBC and Spec pools having the lowest variation within pools followed by Fluor and Volume pooling methods, the main factor in determining the variability or error in animal contribution was the method for measuring DNA concentration. Furthermore, the mechanisms of pool construction such as pipetting, extraction efficiency and sample homogeneity had little impact on variation in animal contributions as evidenced by the tight clustering of the correlation matrix with method; animal contributions among pools within method were highly correlated and less correlated between pooling methods.

Although both are commonly used for DNA pooling construction, a lack of agreement between spectrofluorometry and spectrophotometry methods for measuring DNA concentration has been previously documented (Holden et al., 2009; Li et al., 2014; Yu et al., 2017). Though results from the present study were more favorable for Spec methods over Fluor methods in DNA pooling, caution should be taken before concluding that one DNA quantification method is more accurate for pool construction compared to another, as accuracy of both methods can be influenced by the quality of the DNA present, impurities in the sample, equilibration or mixing of DNA from multiple sources after combining individuals in the pool and structure of the DNA (Li et al., 2014). It is possible that utilizing white blood cell counts yields a more equal sample representation within pools because this approach is based on the relative constant DNA content in individual white blood cells and is not subject to variation that could be introduced based on DNA quantification method. While variation in sample representation between DNA quantification methods has been previously demonstrated, further research is warranted to determine which DNA quantification method is optimal for genotyping, which is beyond the scope of the current study.

Pools constructed based on whole blood volume resulted in greater variability in individual sample representation compared to CBC pools, and had greater variability compared to all other pooling methods. A previous study by Craig et al. (2009) demonstrated that pooling whole blood samples by volume was successful in identifying associated genes in a case/control study. However, the authors acknowledged that pooling whole blood by volume would result in unequal sample representation within pools. Therefore, pooling by blood volume may not be an accurate approach when completing genotyping studies for complex traits, especially disease related traits, because variation in individual white blood cell counts would be expected due to variation in immune response and hydration.

Pooling samples based on equalized white blood cell counts offers many benefits in terms of reduced cost, labor, and time. Large scale association studies typically utilize 100 samples per pool. At a cost of $3 per sample for DNA extraction and $25 per sample for genotyping, the cost to genotype 100 individual samples would cost approximately $2,800. In comparison, pooling genomic DNA reduces the cost of genotyping to $25 for the pool of 100 samples, totaling approximately $325. For samples pooled based on CBC, hematology analysis cost $2 per sample, this combined with genotyping cost results in an estimated total of $228 per pool of 100 animals. Furthermore, extracting DNA from 100 individual samples typically requires 12–16 h depending on the available equipment, while pooling samples based on CBC requires 8–10 h to measure CBC, pool samples, and extract DNA. There are several challenges that should be considered before utilizing this method. First, white blood cell concentration needs to be quantified prior to freezing samples, which requires access to a hematology analyzer. Once frozen, the cells will lyse and white blood cell count can no longer be used to determine DNA concentration. Second, because DNA is not extracted from individual samples, this pooling method prohibits further exploration of individual genotypes. However, this is not necessarily a limiting factor because subsequent stages of genotyping studies are often done using a population independent from the discovery study. Furthermore, not all of the blood collected needs to be pooled. Aliquots of individual blood samples can be stored for later analysis.

## Conclusion

Major factors limiting the ability to complete large-scale genotyping are the expense, labor, and time required to individually genotype many samples. DNA pooling methods can mitigate these limitations as pooling requires fewer genotyping arrays to estimate allele frequencies in groups of individuals. Furthermore, utilizing DNA pooling methods provides the opportunity to incorporate phenotypes from the commercial sector into genomic evaluations. While DNA pooling is an effective way to reduce the cost of genotyping studies, pooling prior to DNA extraction would further minimize the cost, time, and labor associated with extracting DNA from each individual sample. Pooling of individuals based on white blood cell counts provides a more accurate representation of the individuals DNA in the pool. This result suggests an accurate and lower cost approach to pooling samples for genome wide association studies.

## Data Availability Statement

The datasets presented in this study can be found in online repositories. The names of the repository/repositories and accession number(s) can be found below: https://data.nal.usda.gov/dataset/evaluating-accuracy-dna-pool-construction-based-white-blood-cell-counts, dff01bc3-40f4-407a-8bd3-34fa1b 28b2bb.

## Ethics Statement

The animal study was reviewed and approved by U.S. Meat Animal Research Center (USMARC) Institutional Animal Care and Use Committee.

## Author Contributions

All authors contributed intellectually to the design and interpretation of this study. The laboratory work was primarily complete by TM with support from CC-M. Statistical analysis was completed by JK. The manuscript was primarily completed by AA. All authors contributed to the editing of this manuscript.

## Conflict of Interest

The authors declare that the research was conducted in the absence of any commercial or financial relationships that could be construed as a potential conflict of interest.
